# Ventricular Fibrillation Cardiopulmonary Arrest Following Micra™ Leadless Pacemaker Implantation

**DOI:** 10.19102/icrm.2021.121102

**Published:** 2021-11-15

**Authors:** Huzaifa Ahmad, Vijaywant Brar, Nausharwan Butt, Vishaka Chetram, Seth J. Worley, Susan O’Donoghue

**Affiliations:** ^1^MedStar Washington Hospital Center, Washington, DC, USA; ^2^Division of Cardiac Electrophysiology, MedStar Heart and Vascular Institute, Washington, DC, USA

**Keywords:** Cardiac arrest, leadless pacemaker, Micra, ventricular tachycardia

## Abstract

Leadless cardiac pacemakers such as the Micra™ transcatheter leadless pacing system (Medtronic, Minneapolis, MN, USA) are an alternative to traditional transvenous pacemakers. Implantation of leadless pacemakers, albeit safe, may be associated with complications, including cardiac tamponade; high capture thresholds; and, rarely, ventricular arrhythmias. We report a case of ventricular fibrillation arrest following the implantation of a Micra™ leadless pacemaker.

## Introduction

Leadless cardiac pacemakers (PMs) are an alternative to conventional transvenous PMs designed to avoid the need for transvenous leads and pockets.^1^ PM-related adverse events remain substantial; they occur in one in eight patients, despite advancements in pacing technology over the past six decades.^2^ Transvenous PM leads can undergo dislodgment, insulation failure, and may act as a portal for infection into the vascular space, whereas PM pockets are susceptible to hematomas and infections.^3^ Thus, the development of a pacing system foregoing leads and the need for a surgical pocket is a progressive advancement in PM technology. This has been achieved following advances in PM battery, component design, and chemistry that have led to leadless pacemakers (LPs) small enough to be placed directly in the heart, which may lead to a reduction in complications associated with conventional transvenous leads and pockets.^4^

Leadless cardiac PMs are completely self-contained, encapsulated in a small unit, and are affixed by nitinol tines to the myocardium in the right ventricle through a minimally invasive transcatheter approach via the femoral vein. Reynolds et al. and Reddy et al. reported two multicenter studies which found LPs to be a promising alternative to older transvenous systems.^4,5^

The Micra™ transcatheter leadless pacing system (Medtronic, Minneapolis, MN, USA), an example of an LP, has demonstrated high procedural success rates.^6^ However, the procedure may be associated with complications, which include traumatic cardiac injury with cardiac perforation and pericardial effusion; high capture thresholds; and, rarely, ventricular arrhythmias.^7,8^ While some of these complications such as traumatic cardiac injury may be attributed to the learning curve of operators handling this novel technology, other complications such as arrhythmia may be secondary to the implant position of the device.^9^

Few cases of ventricular arrhythmias due to Micra™ implantation have been reported in the literature. We wish to add to the literature by presenting a case of ventricular fibrillation (VF) arrest temporally related to Micra™ implantation.

## Case presentation

**Figure 1:**
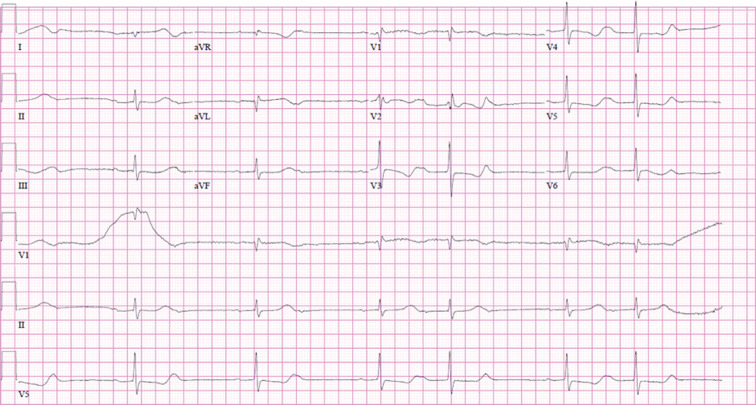
Baseline ECG.

A 62-year-old woman with a history of hypertension, type 2 diabetes, and end-stage renal disease was admitted to the hospital with bleeding from her arteriovenous (AV) graft and was found to have methicillin-resistant *Staphylococcus aureus* bacteremia. The source of her bacteremia was thought to be her AV graft, which was excised for source control. Her initial electrocardiogram (ECG) showed sinus rhythm **(Figure 1)**, but her hospital course was complicated by the development of complete heart block with a narrow junctional escape rhythm and paroxysmal atrial fibrillation. Further workup with a transesophageal echocardiogram demonstrated a small mobile echo density measuring 4 × 5 mm attached to the coumadin ridge. She was placed on prophylactic and not therapeutic anticoagulation due to a high bleeding risk. Cardiac computed tomography ruled out the involvement of the aortic root, ie, aortic root abscess or pseudoaneurysm. No other reversible causes of her complete heart block could be identified. Subsequently, a transvenous pacing wire was successfully placed within the right ventricle.

Given her diagnosis of infective endocarditis and need for a prolonged course of antibiotics, the decision was made to proceed with the implantation of a permanent pacing system. The patient decided to undergo Micra™ transcatheter LP implantation, due to her recent history of extraction of her potentially infected left upper-extremity AV graft and previously documented right subclavian vein occlusion. Subsequently, the patient underwent Micra™ implantation through the right femoral vein. After the device was deployed, the pull-and-hold test was performed to ensure adequate fixation. The postfixation electrical testing of the device demonstrated an R-wave sensing value of 6.5 mV, an impedance of 550 Ω, and a pacing threshold of 1.3 V at 0.24 ms. The latter improved to less than 1 V at 0.24 ms before the end of the case. Hence, the decision was made to cut the tethering suture and release the Micra™ device. Postprocedure, the patient remained stable and was transferred to the medical floor.

**Figure 2:**
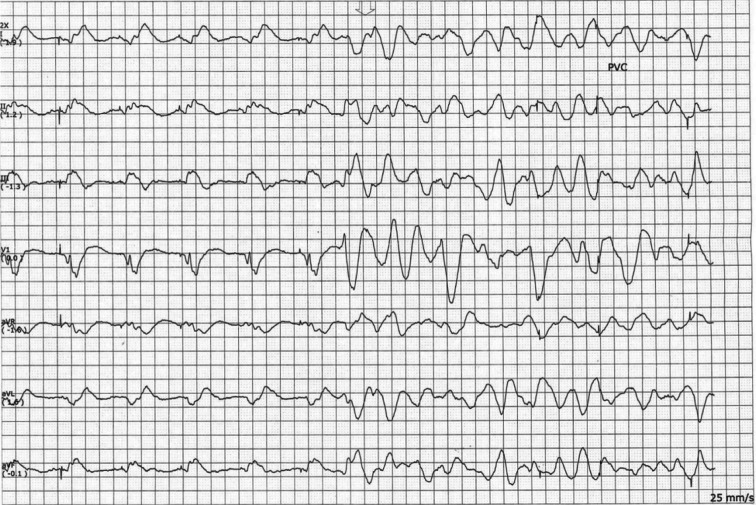
VF following Micra™ implantation.

Approximately five hours following Micra™ insertion, she developed VF cardiac arrest. The telemetry strip for the event is shown in **Figure 2**. She did not have hyperkalemia and was not on any concomitant proarrhythmogenic or QT-prolonging medications. She received seven shocks and eight ampules of epinephrine in addition to intravenous amiodarone and lidocaine boluses. Return of spontaneous circulation was subsequently achieved, and she was transferred to the cardiovascular intensive care unit.

**Figure 3:**
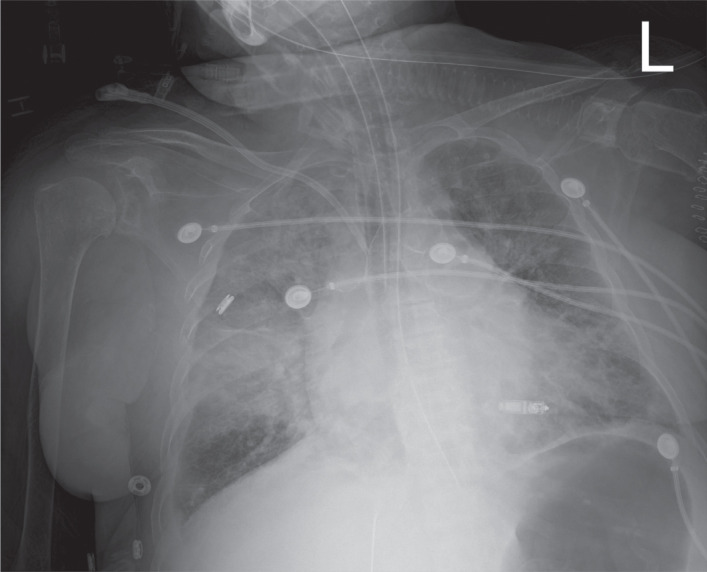
Appropriate Micra™ device position postimplantation.

In the cardiovascular intensive care unit, she was started on norepinephrine and vasopressin for hemodynamic support. Her Micra™ interrogation showed normal device function with stable impedance, sensing, and slightly higher pacing threshold values. The latter, however, remained below the programmed pacing output of the device. A post–cardiac arrest ECG showed a ventricular-paced rhythm with occasional premature ventricular complexes (PVCs). Transthoracic echocardiography did not show any evidence of pericardial effusion, and her chest X-ray showed stable device positioning from implantation and diffuse infiltrates as seen in **Figure 3**. However, the chest X-ray was obtained after the VF arrest and she was not in respiratory distress or hypoxia prior to the arrest. Her serum electrolytes, including potassium, and blood counts were within the normal limits and unchanged from admission. Given the lack of a clear explanation for her VF arrest and given the temporal association with the Micra™ device implantation, her VF was presumed to be secondary to myocardial irritation from the Micra™ device. Unfortunately, she developed worsening shock with increasing vasopressor requirements and was later transitioned to comfort care following a family discussion and subsequently expired within 18 hours of the original procedure.

## Discussion

This case demonstrates potential Micra™ LP–induced VF arrest in our patient. Despite extensive workup, the etiology of VF arrest in our patient remained unclear. Although local myocardial irritation from the implantation of the Micra™ device as a trigger of VF is possible, it is unlikely given the difference in the morphology of the PVC that initiated VF versus paced QRS complexes. Other case reports of ventricular arrhythmias following LP device implantation have also been reported.^9–11^ Our case sheds further light on this complication by adding to the existing literature.

Current studies show the Micra™ LP to have an acceptable safety profile; however, reports of perioperative and postoperative ventricular arrhythmias have been described. Ritter et al. originally described the early performance of Micra™ and its safety profile in a prospective multisite study; among the cohort of 140 patients who underwent Micra™ implantation, three individuals developed ventricular arrhythmias that were not associated with death, reoperation, or hospitalization.^12^ In a retrospective Swiss study evaluating the safety and efficacy of Micra™ implantation in 92 patients, one patient developed unstable ventricular tachycardia (VT) during implantation; two months later, he was hospitalized again with intractable VT requiring ablation. During the ablation, the VT was mapped to an origin site close to the insertion site of the device.^7^ In contrast, early results from the Micra™ Postapproval Registry, an ongoing global, prospective, observational registry evaluating the safety and efficacy of Micra™ implantation, showed that none of the 1,801 patients who underwent the procedure developed ventricular arrhythmias.^13^

Different mechanisms for the development of ventricular arrhythmias after Micra™ implantation have been proposed.^9,11,12^ Amin et al. reported a case of PVC-induced polymorphic VT resulting in episodes of near-syncope and dizziness in a 74-year-old patient one day after Micra™ LP implantation. The PVCs were hypothesized to be secondary to local irritation of the right ventricular myocardium at the site of Micra™ implantation. Similarly, in the Swiss study, the unstable VT was attributed to a proarrhythmic effect of the nitinol fixation tines.^7^ Another report describing polymorphic VT post–Micra™ implantation hypothesized that local inflammation via cytokine-mediated cardiac remodeling likely contributed to VT in their case.^14^ In contrast, Da Costa et al. described a case of cardiac arrest within hours following Micra™ implantation; the ventricular tachyarrhythmia occurred during ventricular pacing and not spontaneously, and pacing-related induction of VF was considered a possibility.^11^ Coronary ischemia may be a possible etiologic factor; however, our patient had no history of coronary artery disease.

Importantly, while some of these cases were managed conservatively, others have shown that retrieval of the LP results in the resolution of the arrhythmias. In the case described by Amin et al., the patient subsequently underwent a second Micra™ implantation with retrieval of the prior device to alleviate symptoms.^9^ A report by Olsen et al. describes cardiac arrest due to VF post–Micra™ implantation that resolved only after the removal of the LP.^10^ Similarly, the case of recurrent hemodynamically unstable VT from the Swiss study demonstrated that, despite management with amiodarone, β-blockers, verapamil, lidocaine, and external defibrillation, the arrhythmia ultimately resolved after surgical explantation of the Micra™ device.^7^ In contrast to these three reports, the case of polymorphic VT after Micra™ implantation was managed with intravenous steroids and overdrive pacing, resulting in the resolution of repolarization abnormalities.^14^

After Micra™ LP implantation, patients can abruptly develop ventricular arrhythmias, leading to significant morbidity and mortality rates. Although the literature is currently limited to case reports following the initial device performance data, we believe that further, larger studies will help to illustrate this relationship. These adverse events highlight the importance of close monitoring of patients in the postoperative period to minimize complications with timely intervention. We propose that patients undergoing Micra™ implantation should be closely monitored for ventricular arrhythmias in the early postoperative period.

## Conclusion

LPs require direct implantation into the myocardium and, though promising, may be associated with life-threatening proarrhythmic effects in some patients. Patients with such devices may benefit from close postprocedural monitoring for arrhythmias and other complications. Larger studies are needed to investigate the possible risk factors for arrhythmias in patients with leadless pacing devices and optimize postprocedural management.
